# Delivering relational continuity of care in UK general practice: a scoping review

**DOI:** 10.3399/BJGPO.2024.0041

**Published:** 2024-06-26

**Authors:** Miglena N Fox, Jon M Dickson, Patrick Burch, Daniel Hind, Olivia Hawksworth

**Affiliations:** 1 Centre for Health and Related Research, University of Sheffield, Sheffield, UK; 2 Medicine Optimisation Team, South Yorkshire Integrated Care Board, SY ICB, Sheffield, UK; 3 Sheffield Centre for Health and Related Research, University of Sheffield, Sheffield, UK; 4 Centre for Primary Care, University of Manchester, Manchester, UK

**Keywords:** general practice, continuity of patient care, primary health care

## Abstract

**Background:**

Relational continuity of care (patients seeing the same GP) is associated with better outcomes for patients, but it has been declining in general practice in the UK.

**Aim:**

To understand what interventions have been tried to improve relational continuity of care in general practice in the UK.

**Design & setting:**

Scoping review of articles on UK General Practice and written in English.

**Method:**

An electronic search of MEDLINE, Embase, and Scopus from 2002 to the present day was undertaken. Sources of grey literature were also searched. Studies that detailed service-level methods of achieving relational continuity of care with a GP in the UK were eligible for inclusion. Interventions were described narratively in relation to the elements listed in the Template for Intervention Description and Replication (TIDieR). A logic model describing the rationale behind interventions was constructed.

**Results:**

Seventeen unique interventions were identified. The interventions used a wide variety of strategies to try to improve relational continuity. This included personal lists, amended booking processes, regular reviews, digital technology, facilitated follow-ups, altered appointment times, and use of acute hubs. Twelve of the interventions targeted specific patient groups for increased continuity while others focused on increasing continuity for all patients. Changes in continuity levels were measured inconsistently using several different methods.

**Conclusion:**

Several different strategies have been used in UK general practices in an attempt to improve relational continuity of care. While there is a similar underlying logic to these interventions, their scope, aims, and methods vary considerably. Furthermore, owing to a weak evidence base, comparing their efficacy remains challenging.

## How this fits in

There have been a variety of interventions aimed at improving relational continuity of care in NHS general practice. Using the TIDieR framework, this scoping review provides a breakdown of the different strategies employed throughout the UK. While there was insufficient data available to directly compare the efficacy of different interventions, this work provides a synthesis of what has been tried. These results and analysis highlight that the evidence base for delivery of relational continuity is weak but serve as a useful foundation on which to base policy, quality improvement interventions, and future research.

## Introduction

Relational continuity of care, the ongoing relationship between a patient and a clinician, is regarded as a distinguishing feature of general practice and is valued by GPs as one of the core aspects of their role.^1,2^ Provision of relational continuity has been associated with a range of desirable clinical outcomes and reductions in healthcare costs.^3–8^ It has been proposed as the driver of these outcomes via a number of mechanisms.^9,10^

Despite evidence of its benefits and its popularity with patients and doctors, relational continuity of care in NHS general practice has been declining.^11,12^ This is likely owing to increasing size of GP practices, changes in staffing and working practices, increased demand, and increased patient expectations.^13,14^ A call to reverse this decline has been made by multiple professionals, patients, professional groups, and a recent parliamentary select committee.^7,12,15^ However, it is unclear how best to do this reversal. Some advocate a return to a ‘traditional’ type of system, where each patient has a named doctor who they see whenever possible.^9^ Others contend that this is not a practical solution for all practices and that continuity should be focused on patients who are deemed to need it the most.^16,17^ The Royal College of General Practitioners (RCGP) has designed a toolkit for practices to improve relational continuity but recognises there is unlikely to be a one-size-fits-all solution.^15^ We could find no publications synthesising approaches taken to improve continuity in UK general practice.

In this paper, we present a scoping review of studies describing methods of delivering relational continuity of care in NHS general practice.^18^ Our objectives were to: (1) search for evidence on methods of delivering relational continuity in NHS general practice in the UK; (2) build an overview of the existing research; (3) identify knowledge gaps; and (4) inform opportunities for future research.

## Method

This review was conducted in line with the Joanna Briggs Institute (JBI) methodology for scoping reviews^19^ and is reported according to the Preferred Reporting Items for Systematic Reviews and Meta-Analyses extension for Scoping Reviews (PRISMA-ScR) statement.^20^ The protocol was set before conducting the review.

### Eligibility criteria

The scope of our review is structured around the Population (or participants), Context, Concept formula.^21^ Eligible populations were those registered with UK general practices or other primary care settings (walk-in centres and community clinics, non-primary care settings, or from outside of the UK were ineligible). The context of eligible articles was general practices and GPs. Studies of hospital and inpatient care, surgical aftercare, and studies that were not primarily about GPs were excluded. To be eligible in terms of concept, a study had to present applied case studies detailing service-level methods and/or mechanisms of achieving relational continuity of care. Studies with educational components were included only where they were quality improvements focused on improving relational continuity. Observational studies of associations between continuity of care and clinical outcomes, patient preference studies, discursive articles, review articles, and letters were ineligible. Articles published before 2002 or in languages other than English were excluded.

### Information sources and search strategy

We searched MEDLINE, Embase, Overton, and Scopus applying limits such that only English language articles and those published since 2002 were retrieved. The full MEDLINE and Embase search strategies are provided in Supplementary Information S1. We searched Overton using a reference tracking method whereby the first 10 relevant policy documents were screened for references that might be suitable for inclusion.^22^ We searched OpenGrey, the King’s Fund, Nuffield Trust, The Health Foundation and undertook a Google search for grey literature. The search strategies are outlined in Supplementary Information S1. All searches were undertaken in March 2023 (MEDLINE: 13 March 2023; Embase: 28 March 2023; Overton and Scopus and grey literature: 18 March 2023).

### Selection of sources of evidence

All search results were uploaded to Rayyan^23^ and duplicates were removed. The title and abstract of each result was screened against the eligibility criteria by at least two reviewers. Where eligibility was unclear, the full text was sought for retrieval.

### Data charting process and data items

Data charting forms were created and piloted in Google Sheets. The data items charted were the items from the Template for Intervention Description and Replication (TIDieR) checklist,^24^ which were supplemented by elements of the Dorling *et al* checklist.^25^ These were as follows: the rationale of the essential elements of the intervention (’why’); what materials were used; what procedures; who provided the intervention; how the intervention was delivered; where the intervention occurred; when and how much; whether personalisation of the intervention was planned (’tailoring’); details of any modifications during the course of the study; fidelity, how well planned and how well delivered. We also extracted relational continuity index outcomes and research gaps.

The taxonomy from Expert Recommendations for Implementing Change (ERIC) was utilised to assess the implementation strategies of health innovations into standard care.^26^

### Synthesis of results

We produced narrative and tabular summaries, as a well as a programme theory model (logic model), showing how authors intended that the intervention procedures would affect outcomes.

## Results

### Selection of sources of evidence

**Figure 1.**
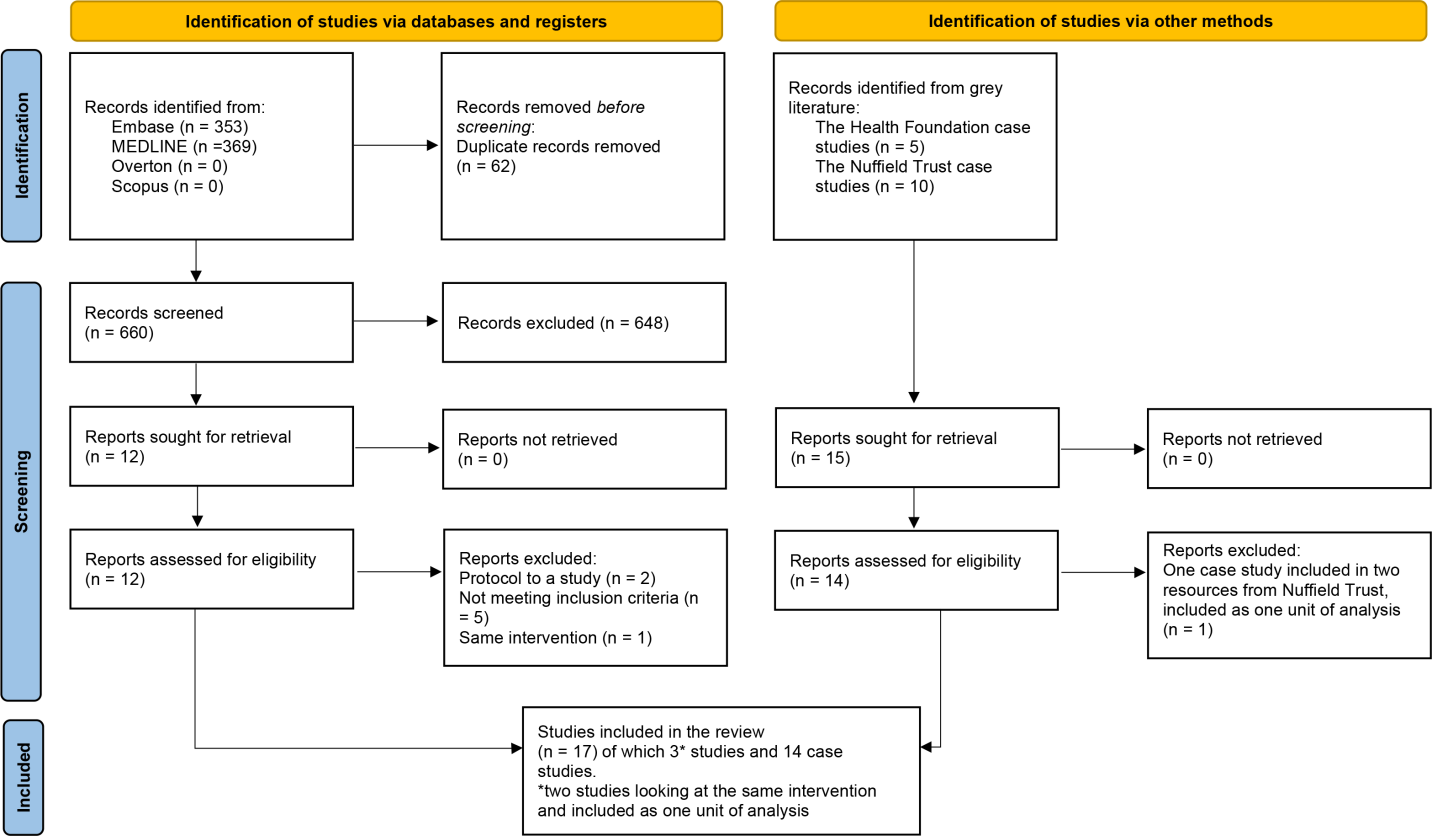
PRISMA flow chart

Database searches identified 660 records after the removal of duplicates (Figure 1). Twelve records underwent full-text screening, at which stage eight were excluded (Supplementary Information S2, Supplementary Table S12) and four were eligible for inclusion.^27–30^ Two records referred to the same intervention so were treated as one unit of analysis;^27^,^28^ thus, three unique intervention models were identified. Grey literature searches yielded three records^31–33^ reporting 15 interventions. One case study was included in both Nuffield Trust papers from 2019^33^ and 2022,^32^ hence was included as one unit of analysis resulting in total of 14 case studies included. In total, 17 unique interventions were identified.

### Characteristics of sources of evidence

#### Study and intervention characteristics and study rationale

The 17 studies took place in different locations in the UK. The study sites served populations ranging in size from 1546–420 000 patients, with different characteristics; for example, age, rurality. See Table 1 for full list of study characteristics.

**Table 1.** Location and population characteristics

Study ID	Where	Population	Relevant population characteristics
Tammes, 2019, Barker 2016(cohorts)^27,28^	England (Tammes: 139 English GP practices), (Barker: 200 general practices)	Tammes: a random sample of 27 500 patientsBarker: 255 469 patients	Tammes: patients who were aged 65–84 years in 2012Barker: patients aged between 65 and 85, after excluding those aged 75.
Slater, 2021(mixed-methods study)^29^	Scotland	4000 patients	Deprived area
Salisbury, 2019(randomised controlled trial)^30^	33 general practices located in three areas of England and Scotland: Manchester, Bristol, and Ayrshire and Arran	A total of 1546 patients were enrolled in the study, with 797 patients assigned to the 3D intervention from 16 practices, and 749 patients assigned to usual care from 17 practices	A diverse range of locations, encompassing both affluent and deprived areas, as well as rural, urban, and suburban areas
The Health Foundation, 2022, five case studies: Continuity counts^31^	One practice was located in Exmouth, one in mid-Devon (rural location), and the other three in Exeter	Total population of 41 129 people	No data
The Health Foundation, 2022, five case studies: Morecambe Bay Primary Care Collaborative (MBPCC)^31^	10 practices in South Cumbria and Morecambe Bay	Population of 97 275	No data
The Health Foundation, 2022, five case studies; One care^31^	23 practices in North Somerset, South Gloucester, and Bristol	Population of around 400 000 patients	Both deprived and affluent backgrounds, as well as individuals from rural and urban environments
The Health Foundation, 2022, five case studies: Continuity by design (Pier Health Partnership)^31^	Weston and Worle locality in the South West of England	Population of 94 000 patients	Weston-super-Mare is recognised for its challenges related to GP shortages, large patient lists, high patient demand, significant workload, and ongoing difficulties with GP recruitment
The Health Foundation, 2022, five case studies: Valentine Health Partnership^31^	Woolwich, South East London	Population of >26 000 patients	Younger and ethnically diverse transient population. The population of this partnership is changing often and is increasingly socioeconomically diverse
Nuffield Trust, 2022, four case studies: AT Group Digital Hub^32^	Greater London	Total registered population of 420 000 patients	No data
Nuffield Trust, 2022, four case studies: St Austell Healthcare^32^	Five sites in St Austell, Cornwall	Population of 36 800 patients	Mainly urban or suburban areas, including one rural and deprivation group 5 with a high levels of chronic disease
Nuffield Trust, 2022, four case studies: Quay Health Solutions^32^	North Southwark, London	Population of 200 000 patients	Top two deprivation decile
Nuffield Trust, 2022, four case studies: Foundry Healthcare^32^ Nuffield Trust, 2018, evidence review, Lewes, East Sussex^33^	Lewes, East Sussex	Population of 28 200 across five sites	Urban and rural communities with deprivation decile 8
Nuffield Trust, 2018, evidence review, Fleetwood, case study^33^	3 GP practices in town of Fleetwood	Around 30 000 patients	No data
Nuffield Trust, 2018, evidence review, Larwood and Bawtry, case study^33^	Larwood, 5 sites	Population of 32 800 patients	No data
Nuffield Trust, 2018, evidence review, Southampton case study^33^	26 GP practices in Southampton	269 000 patients	No data
Nuffield Trust, 2018, evidence review, Richmond, case study^33^	28 GP practices in Richmond, London	215 000 population	No data
Nuffield Trust, 2018, evidence review, Littlehampton, case study^33^	The Park surgery in Littlehampton	Population of 10 000	High proportion of older people

Although all studies had an element focusing on relational continuity, the underlying rationale for the studies differed and could be fitted into one of five of the following categories: reducing unplanned hospitalisation; improving access while maintaining continuity; providing a named GP; improving outcomes; and providing continuity for reviews. See Supplementary Table S1 for more details.

### Interventions

We categorised the interventions as either a clinical intervention or a service implementation.

### Clinical interventions

#### Assigning patients to clinicians

This occurred in seven studies. Five studies assigned patients to usual or named GPs (*n* = 5).^28,31^ Personal lists were utilised in one study (*n* = 1).^31^ One study examined the NHS policy change introduced in April 2014, which mandated offering patients aged ≥75 years a named, accountable GP (*n* = 1).^27,28^

#### Changing booking processes

This occurred in 13 studies. Nine studies used triage or clinical workstreams to book patients into acute or ongoing care (*n* = 9).^32,33^ One intervention booked ‘tagged patients’ (patients identified as needing continuity) with their usual GP (*n* = 1) (^30^, Valentine Health Partnership). One practice booked all clinical workstreams (usual and acute care) with the usual clinician (*n* = 1) (^30^, Pier Health Partnership). One intervention booked patients with multimorbidity with a named GP (*n* = 1)^30^ and one organised follow-up bookings for patients after an initial consultation (*n* = 1).^29^

#### Offering comprehensive review with GP

One intervention used 6 monthly comprehensive review with the same clinician in order to improve relational continuity (*n* = 1).^30^

#### Patient profiling and identifying patients perceived to benefit most from continuity

**Figure 2.**
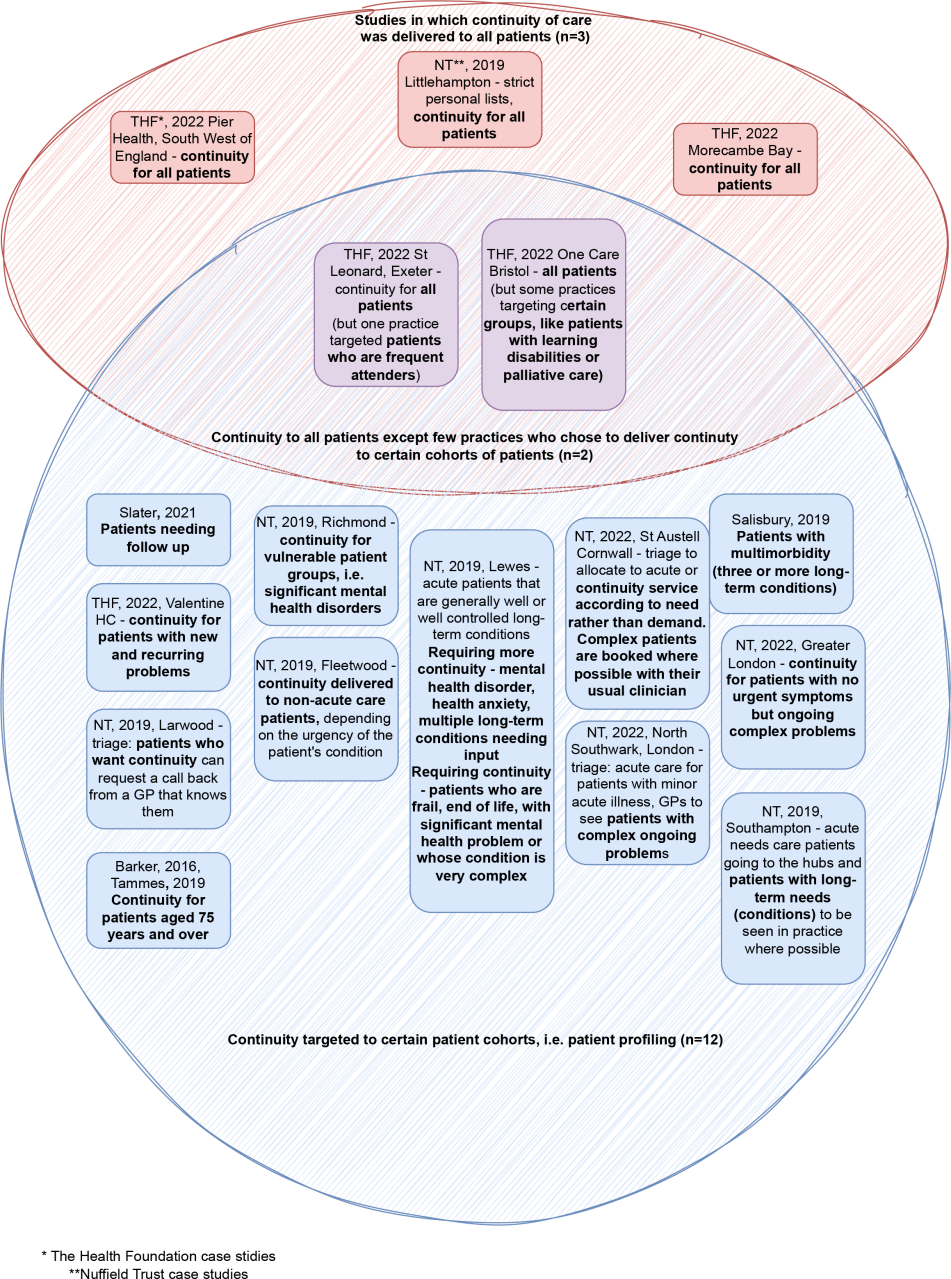
Patient profiling. NT = Nuffield Trust; THF = The Health Foundation

Three interventions delivered continuity to all patients.^31,33^ Twelve studies used patient profiling to identify patients expected to benefit more from continuity.^27–33^ Two studies did a mixture of both.^31^ Figure 2 presents the results of patient profiling.

#### Introduction of digital technology

Technological interventions were used in 10 studies (*n* = 10).^30–32^ This included online consultations, digital bookings and self-help tools, training algorithms and tools, prompts and reminders, and results delivery.

#### Facilitate follow-ups

Follow-ups with the same clinician were offered to patients who were informed of test results, started new medications, or after acute illness (^28^,^32^ Littlehampton) (*n* = 2). One study looked at patients with increased GP consultations in the past 6 months and ensured that these patients were booked with the same GP (^30^, Valentine Health Partnership) (*n* = 1).

#### Increased number of appointments and acute hubs

Ten interventions expanded appointment availability or extended access beyond regular surgery hours.^29,32,33^ These additional appointments, often facilitated through acute hubs and out-of-hours services, aimed to take the acute care out of regular surgery hours and thus free GPs to deliver continuity. Eight interventions used acute hubs with supplementary appointments^32,33^ (*n* = 8). One intervention added telephone and online services to increase appointment capacity (^32^, Larwood) (*n* = 1). Another intervention introduced shorter pre-bookable follow-up appointments, attempting to optimise consultation efficiency^29^ (*n* = 1). Further details on clinical interventions are in Supplementary Table S2.

### Service and implementation interventions

**Figure 3.**
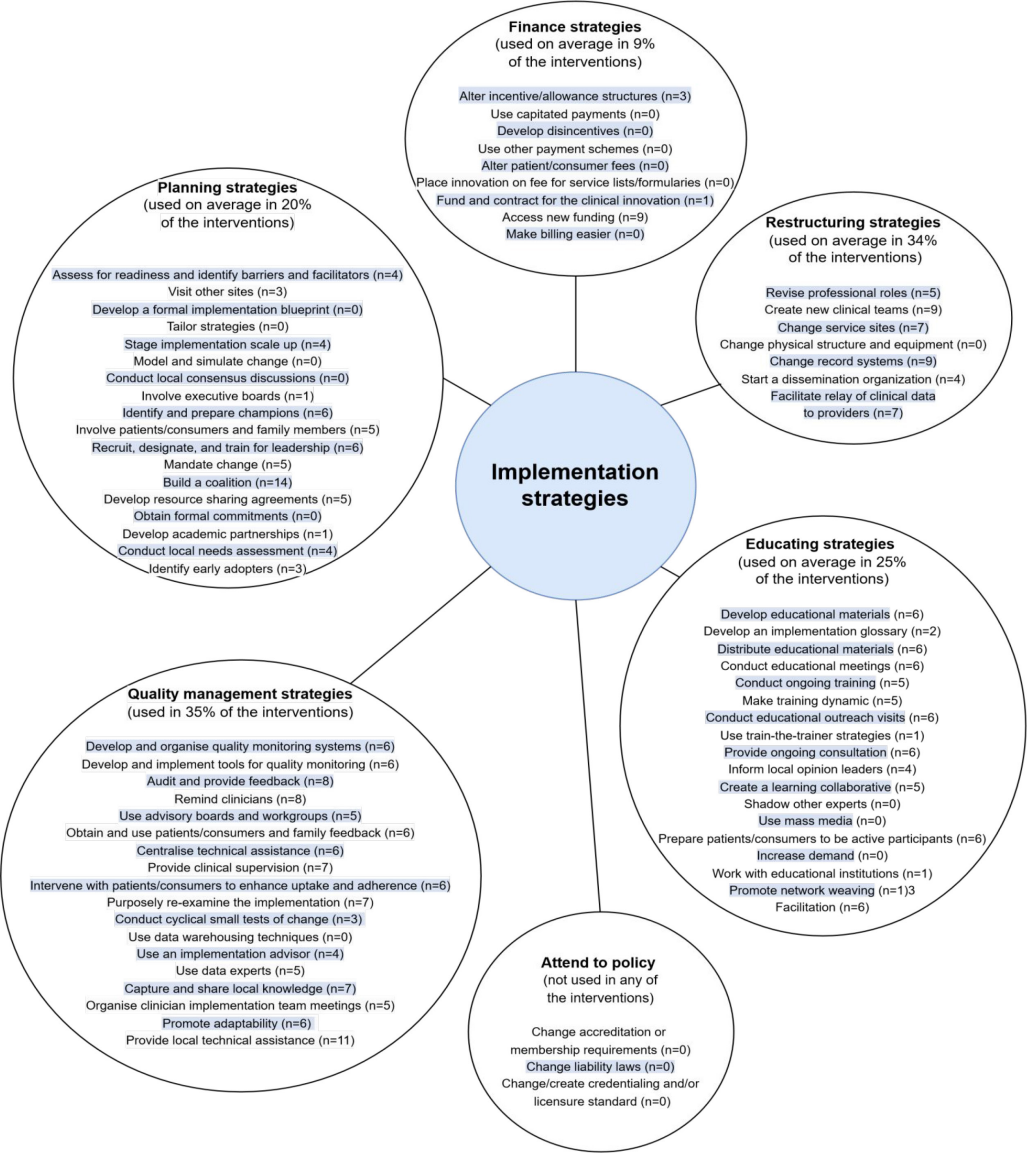
Implementation strategies. Please note, ‘*n*’ represents the number of studies the said implementation strategy was used in, that is, if *n* = 0, it means that the said strategy was not used in any of the interventions. The pale blue highlighting is solely to help with the readability and segregation of the individual strategies.

The taxonomy from Expert Recommendations for Implementing Change (ERIC) was utilised to assess the implementation strategies of health innovations into standard care.^26^ Service and implementation interventions fitted into the following six categories: planning strategies; educating strategies; finance strategies; restructuring strategies; quality management strategies; and attend to policy. Each strategy and the number of studies (*n*) it was used are detailed in Figure 3.

#### Materials used in the interventions

Four of the studies used letters, written care plans or business cards, informing patients of their named GP and reminding patients to book with them whenever possible (*n* = 4). Digital prompts and resources were used in 10 studies.^30–32^ Five studies used educational materials, leaflets, posters, and slides.^30,31^ Five studies used reference documents, toolkits and protocols or scripts to help with training, booking and delivery of interventions.^30,31^ More detail is in Supplementary Table S3.

#### Who provided the intervention

Providers were divided into the following three main groups: non-clinical practice staff; clinical staff (GPs, nurses, and so on); and the research and implementation team (project managers, data analysts, and so on). The results are in Supplementary Table S4.

#### How the intervention was delivered

Most interventions (*n* = 12) were delivered face to face (*n* = 1;^29^ *n* = 1;^30^ *n* = 4;^31^ *n* = 2;^32^ and *n* = 4^33^). Some reported multiple delivery methods such as face to face, online, or telephone. Telephone consultations were employed in nine interventions (*n* = 1;^29^ *n* = 1;^31^ *n* = 3;^32^ and *n* = 4^33^), while online means were utilised in eight (*n* =;^29^ *n* =1;^31^ *n* =3;^32^ and *n* =4^33^). Three used letters, cards, or emails (*n* = 1;^30^ and *n* = 2^31^). Five interventions also employed group delivery such as workshops and webinars (*n* =5^31^). Two articles lacked clarity on intervention delivery (*n* =1;^31^ *n* = 1 ^33^). Supplementary Table S5 gives further details.:

#### Other data items

Additional data items on tailoring (Supplementary Table S7), modification (Supplementary Table S8), fidelity (Supplementary Table S9), and frequency of intervention delivery (Supplementary Table S6) are available in the supplementary files. Outcome data and identified research gaps are listed in Supplementary Tables S10 and S11.

### Synthesis of results

**Figure 4.**
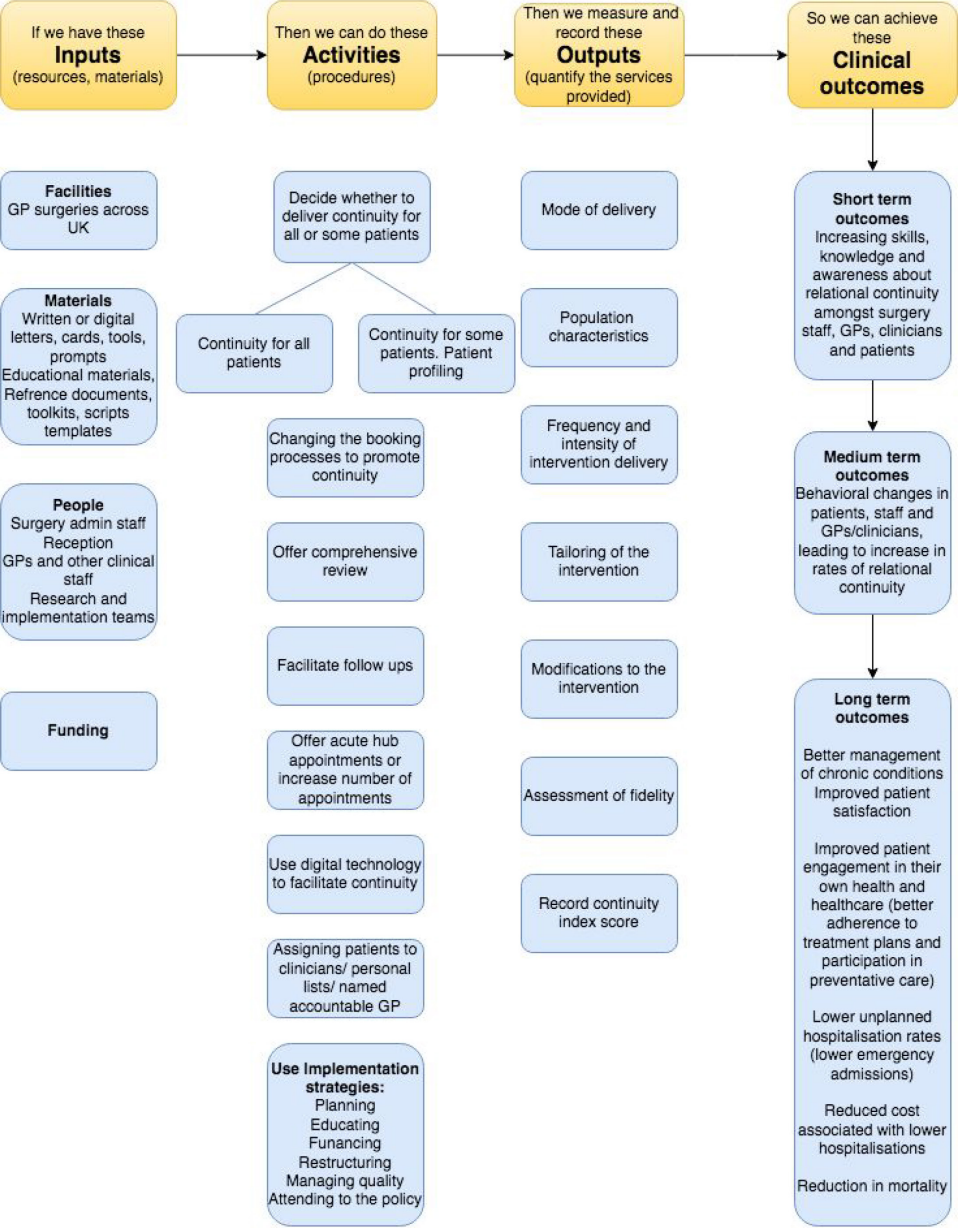
Programme theory model

To summarise the results and illustrate the important findings, a programme theory (logic model) was developed. A logic model is a technique used to illustrate certain components of the programme theory usually presented in a linear sequence, and incorporating the mechanisms through which an intervention is believed to produce specific outcomes.^34^ Logic models are used to help understand the important features of a programme and aid the description of what might work best when it comes to achieving a certain goal, in this case, relational continuity in general practice. This is represented in Figure 4.

## Discussion

### Summary

To our knowledge, this is the first scoping review focusing on the methods used to deliver relational continuity of care in general practice in the UK. We identified 17 interventions using a variety of strategies with a range of complexity. Common strategies involved altering booking processes, assigning patients to clinicians, and using digital technology to promote continuity. Interventions varied in terms of whether they were aiming to improve continuity of care for all patients or for specific groups. Our synthesis of the results of these studies provides a useful breakdown and typology of potential interventions on which to base policy, quality improvement interventions, and research.

### Strengths and limitations

The majority of interventions were found in grey, rather than peer-reviewed literature. Several included limited descriptions of interventions, and outcome measures were often not reported. It was not possible to compare the efficacy of interventions and identify which were most effective because of limited reporting of outcomes and the greater part of the studies being uncontrolled, single-arm designs. This paper focuses on the UK because international health systems differ considerably and may not be applicable to each other. Nevertheless, a review of international efforts to improve continuity may also be useful.

### Comparison with existing literature

The associations between relational continuity and multiple health outcomes are well established.^5,9^ There is evidence^35^ and plausible mechanisms as to why this relationship is likely to be causal.^9^ We understand clinician and patient perspectives on continuity,^2,36,37^ and there is now organisational and some political will to improve continuity.^12,16^

### Implications for research and practice

This review distils existing knowledge and practices aimed at achieving continuity and serves as a valuable starting point for those aiming to improve continuity. It can be used as an adjunct to existing resources, such as the RCGP toolkit,^15^ to enable quality improvement work, as well as providing a framework for considering future research or interventions.

Our reporting of results was limited by the quality of the retrieved literature and highlighted the impossibility of directly comparing the efficacy of existing interventions to one another using reported data. We recommend that any future interventions to improve continuity are reported using a recognised framework (such as TIDieR). While we would caution researchers from trying to directly compare the efficacy of interventions, we would recommend the recording and reporting of continuity levels using recognised measurements such as Usual Provider of Care (UPC) index or St Leonard Index of Continuity of Care (SLICC).^38^

For many GP practices, delivering continuity is something they are doing on a day-to-day basis. These methods of delivering continuity are going undocumented and are not captured in the literature. A large project has recently been funded to carry out an assessment of how practices with good relational continuity operate.^39^ Future research should include consideration of trials to improve continuity alongside economic evaluations. These trials are already happening outside the UK.^40^

The current direction of travel in England is to try to improve relational continuity for those who ‘need it’ rather than provide continuity for all.^16,41^ Several of the interventions reviewed in this scoping review used such a strategy and there are lessons to be learnt from their experience. While there appears to be groups of patients who may logically benefit more from continuity (for example, older patients, those with complex multimorbidity) the evidence on the differential benefits of continuity to different patient groups has not, to our knowledge, been synthesised.

Improving relational continuity should be a key priority for NHS general practice. Whether this will happen and whether it will be through a top-down centrally rolled out initiative or through individual practices, primary care networks (PCNs) or integrated care boards (ICBs) is unclear. However, we agree with Gray *et al* and the Health and Social Care Committee that national measurement of continuity will be needed.^12,38^ While we do not think that practices should delay quality improvement measures to try and improve continuity, any large-scale interventions need to be evidenced based, effective, and sensitive to local context. The results of this review show that while we understand what can be done, and may be effective in certain contexts, more research is still required.

## Data

The dataset relied on in this article is available from the corresponding author on reasonable request.
